# Epidemiological Pathology of Dementia: Attributable-Risks at Death in the Medical Research Council Cognitive Function and Ageing Study

**DOI:** 10.1371/journal.pmed.1000180

**Published:** 2009-11-10

**Authors:** Fiona E. Matthews, Carol Brayne, James Lowe, Ian McKeith, Stephen B. Wharton, Paul Ince

**Affiliations:** 1 Medical Research Council (MRC) Biostatistics, University of Cambridge, Cambridge, United Kingdom; 2 Institute of Public Health, University of Cambridge, Cambridge, United Kingdom; 3 Department of Neuropathology, University of Nottingham, Nottingham, United Kingdom; 4 Institute for Ageing and Health, University of Newcastle upon Tyne, Newcastle upon Tyne, United Kingdom; 5 Department of Neuroscience, University of Sheffield, Sheffield, United Kingdom; Mount Sinai School of Medicine, United States of America

## Abstract

**Background:**

Dementia drug development aims to modulate pathological processes that cause clinical syndromes. Population data (epidemiological neuropathology) will help to model and predict the potential impact of such therapies on dementia burden in older people. Presently this can only be explored through post mortem findings. We report the attributable risks (ARs) for dementia at death for common age-related degenerative and vascular pathologies, and other factors, in the MRC Cognitive Function and Ageing Study (MRC CFAS).

**Methods and Findings:**

A multicentre, prospective, longitudinal study of older people in the UK was linked to a brain donation programme. Neuropathology of 456 consecutive brain donations assessed degenerative and vascular pathologies. Logistic regression modelling, with bootstrapping and sensitivity analyses, was used to estimate AR at death for dementia for specific pathologies and other factors. The main contributors to AR at death for dementia in MRC CFAS were age (18%), small brain (12%), neocortical neuritic plaques (8%) and neurofibrillary tangles (11%), small vessel disease (12%), multiple vascular pathologies (9%), and hippocampal atrophy (10%). Other significant factors include cerebral amyloid angiopathy (7%) and Lewy bodies (3%).

**Conclusions:**

Such AR estimates cannot be derived from the living population; rather they estimate the relative contribution of specific pathologies to dementia at death. We found that multiple pathologies determine the overall burden of dementia. The impact of therapy targeted to a specific pathology may be profound when the dementia is relatively “pure,” but may be less impressive for the majority with mixed disease, and in terms of the population. These data justify a range of strategies, and combination therapies, to combat the degenerative and vascular determinants of cognitive decline and dementia.

*Please see later in the article for the Editors' Summary*

## Abstract 2

Researchers from the Medical Research Council Cognitive Function and Ageing Neuropathology Study carry out an analysis of brain pathologies contributing to dementia, within a cohort of elderly individuals in the UK who agreed to brain donation.

## Editors' Summary

**Background:**

Losing one's belongings and forgetting people's names is often a normal part of aging. But increasing forgetfulness can also be a sign of dementia, a group of symptoms caused by several disorders that affect the structure of the brain. The commonest form of dementia is Alzheimer disease. In this, protein clumps called plaques and neurofibrillary tangles form in the brain and cause its degeneration. Vascular dementia, in which problems with blood circulation deprive parts of the brain of oxygen, is also common. People with dementia have problems with two or more “cognitive” functions—thinking, language, memory, understanding, and judgment. As the disease progresses, they gradually lose their ability to deal with normal daily activities until they need total care, their personality often changes, and they may become agitated or aggressive. Dementia is rare before the age of 65 years but about a quarter of people over 85 years old have dementia. Because more people live to a ripe old age these days, the number of people with dementia is increasing. According to the latest estimates, about 35 million people now have dementia and by 2050, 115 million may have the disorder.

**Why Was This Study Done?:**

There is no cure for dementia but many drugs designed to modulate specific abnormal (pathological) changes in the brain that can cause the symptoms of dementia are being developed. To assess the likely impact of these potentially expensive new therapies, experts need to know what proportion of dementia is associated with each type of brain pathology. Although some brain changes can be detected in living brains with techniques such as computed tomography brain scans, most brain changes can only be studied in brains taken from people after death (post mortem brains). In this study, which is part of the UK Medical Research Council Cognitive Function and Ageing Study (MRC CFAS), the researchers look for associations between dementia in elderly people and pathological changes in their post mortem brains and estimate the attributable-risk (AR) for dementia at death associated with specific pathological features in the brain. That is, they estimate the proportion of dementia directly attributable to each type of pathology.

**What Did the Researchers Do and Find?:**

Nearly 20 years ago, the MRC CFAS interviewed more than 18,000 people aged 65 years or older recruited at six sites in England and Wales to determine their cognitive function and their ability to deal with daily activities. 20% of the participants, which included people with and without cognitive impairment, were then assessed in more detail and invited to donate their brains for post mortem examination. As of 2004, 456 individuals had donated their brains. The dementia status of these donors was established using data from their assessment interviews and death certificates, and from interviews with relatives and carers, and their brains were carefully examined for abnormal changes. The researchers then used statistical methods to estimate the AR for dementia at death associated with various abnormal brain changes. The main contributors to AR for dementia at death included age (18% of dementia at death was attributable to this factor), plaques (8%), and neurofibrillary tangles (11%) in a brain region called the neocortex, small blood vessel disease (12%), and multiple abnormal changes in blood vessels (9%).

**What Do These Findings Mean?:**

These findings suggest that multiple abnormal brain changes determine the overall burden of dementia. Importantly, they also suggest that dementia is often associated with mixed pathological changes—many people with dementia had brain changes consistent with both Alzheimer disease and vascular dementia. Because people with dementia live for variable lengths of time during which the abnormal changes in their brain are likely to alter, it may be difficult to extrapolate these findings to living populations of elderly people. Furthermore, only a small percentage of the MRC CFAS participants have donated their brains so the findings of this study may not apply to the general population. Nevertheless, these findings suggest that the new therapies currently under development may do little to reduce the overall burden of dementia because most people's dementia involves multiple pathologies. Consequently, it may be necessary to develop a range of strategies and combination therapies to deal with the ongoing dementia epidemic.

**Additional Information:**

Please access these Web sites via the online version of this summary at http://dx.doi.org/10.1371/journal.pmed.1000180.

- The US National Institute on Aging provides information for patients and carers about forgetfulness and about Alzheimer disease (in English and Spanish)
- The US National Institute of Neurological Disorders and Stroke provides information about dementia (in English and Spanish)
- The UK National Health Service Choices Web site also provides detailed information for patients and their carers about dementia and about Alzheimer disease
- MedlinePlus provides links to additional resources about dementia and Alzheimer disease (in English and Spanish)
- More information about the UK Medical Research Council Cognitive Function and Ageing Study (MRC CFAS) is available

## Introduction

Assessment of brain pathology in the consensus protocols for pathological diagnosis of dementia has been based on semiquantitative methods [1]–[3]. These protocols aspire to distinguish demented and nondemented individuals using thresholds of plaques, neurofibrillary tangles (NFTs), infarcts, and Lewy bodies, so that pathology becomes the “gold standard” for diagnosis. This approach has progressed understanding of clinical phenotypes, genetics, biochemistry, and molecular pathogenesis associated with cognitive decline in older people. Trials of disease modifying therapies are already in progress and proponents of a vascular basis for cognitive dysfunction propose secondary prevention strategies in older people [4]. The scale of the clinical and social problem presented by dementia in ageing populations presents an urgent need to assess the likely impact and cost effectiveness of new, potentially expensive, therapies, and to develop robust biomarkers for diagnosis and progression. Understanding the population impact of therapies that modify the pathobiology of dementia requires an understanding of the burden of cognitive dysfunction directly attributable to a particular pathology. Recently reported trials in Alzheimer disease (AD), alleging divergent outcomes for inhibition of amyloid gamma-secretase and tau aggregation, exemplify this need [5],[6]. These are issues about which conventional case-control cohorts and studies in secondary referral populations, memory clinics, or community volunteers are less informative than the population approach used here. Selection biases are associated with non–population-based studies of older people and lead to unknown effects so that their conclusions may not generalise to the whole population.

Dementia is associated with a high prevalence of mixed Alzheimer, vascular, and other pathologies, and the thresholds of severity that clearly distinguish between an AD brain and the brain of a nondemented individual only capture around 20% of demented people [7]–[9]. True population-based studies of dementia combining longitudinal assessment of clinical state with post mortem brain donation are rare but offer the only means at present of investigating the population-level impact of pathology on cognition [10]. The Cognitive Function and Ageing Study (CFAS) autopsy donor cohort is now of sufficient size to facilitate true “epidemiological neuropathology.” Here we present estimates of the attributable risk of dementia at death associated with specific neuropathological features in this cohort.

## Methods

### Ethics Statement

All procedures received approval from a multicentre Research Ethics Committee. MRC Cognitive Function and Ageing Study (MRC CFAS) is a population-based longitudinal study of people, in their 65th year and over, enrolled from the population-based registers of primary care physicians in six sites in England and Wales [11]. In 1990 these registers provided full geographical population coverage including people living in institutional settings.

In each of five centres random samples generated a recruited cohort of 2,500 individuals per centre (82% response rate) with equal numbers below and above 75 y. Trained interviewers conducted interviews with participants, including basic sociodemographic questions, cognitive examination, and items from the Geriatric Mental State (GMS) organicity scale, activities of daily living, physical health, and medication (see www.cfas.ac.uk).

**Figure 1:**
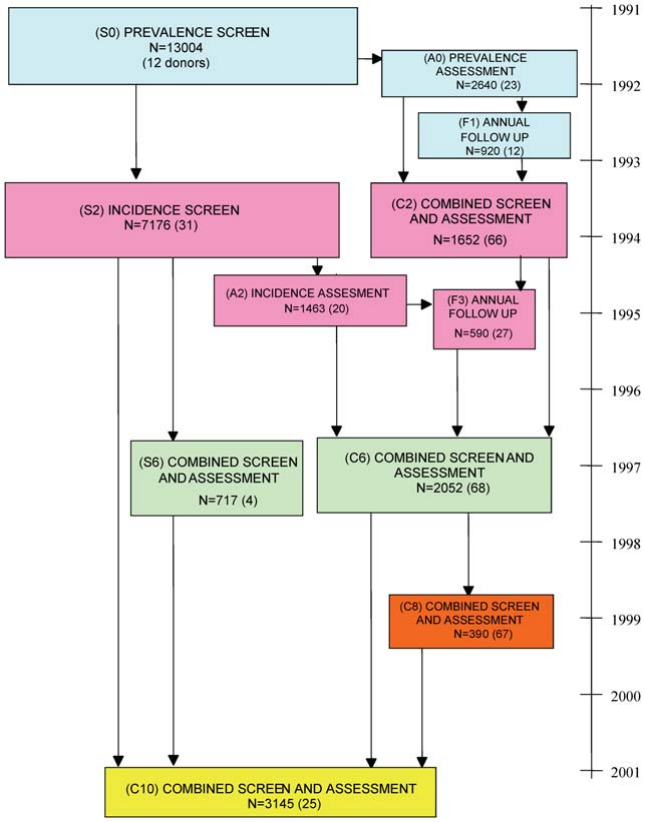
MRC CFAS design. Numbers of interviews and donations from interview waves.

A 20% stratified sample underwent detailed assessment (GMS [12], augmented CAMCOG [13]) repeated after 2 y. This assessment group included those individuals with cognitive impairment and a random sample from the same centre. There were two re-interview cycles of all survivors and several follow-ups in the assessment group only. Study flow is shown in Figure 1. Diagnoses were made using the validated AGECAT algorithm [14].

**Figure 2:**
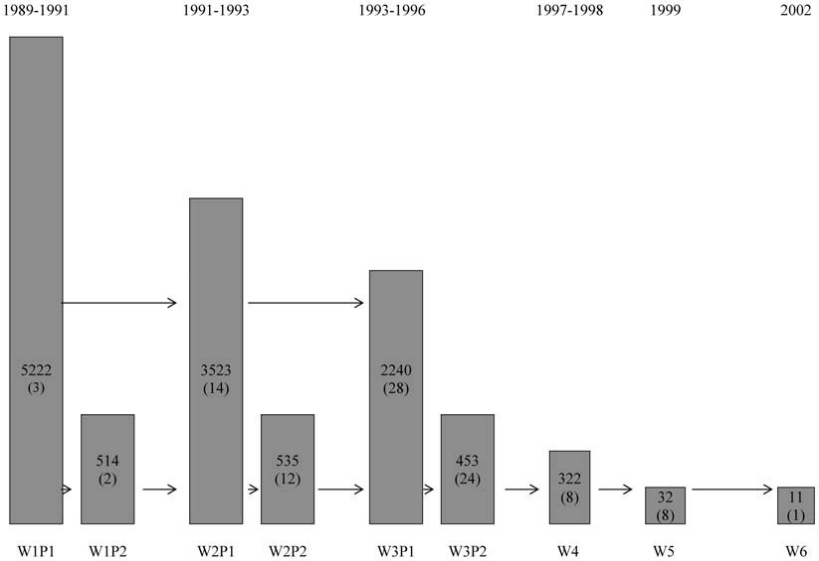
MRC Alpha Study (Liverpool) design. Numbers of interviews and donations from each interview wave.

In the sixth centre (Liverpool) 5,200 people aged 65 y and over, in equal numbers across 5-y bands, were recruited with a population sampling base. This study (ALPHA) started before the other sites but its design and methods enable it to be integrated into CFAS. Interviews in ALPHA were based on GMS and study flow is shown in Figure 2.

Individuals, families, and carers in the assessment group were approached by trained liaison officers and invited to participate in counselling around brain donation. Those who agreed to brain donation were provided with information to allow staff or family involved in the final illness to notify the death and initiate brain donation. Donations still proceeded, wherever possible, for cases coming to autopsy under the coroner.

There were 456 individuals in this analysis, representing all completed brain donations before 1st August 2004. The sample includes 207 individuals previously described (two families contributing to that previous cohort subsequently revoked consent and the data were removed) [7].

Dementia status was established using multiple information sources including AGECAT, notification of dementia in death certificates, a retrospective informant interview (RINI; www.cfas.ac.uk) with relatives and carers after death, and the probability of being demented before death from a Bayesian analysis of all individuals modelling the prevalence and incidence of dementia in CFAS. We could not assign dementia status in 30 individuals in whom the study diagnosis was “not dementia.” These respondents were not included in the analysis because their last interview was more than 6 mo before death, no RINI was available, and dementia was not mentioned on the death certificate.

The neuropathology protocol used standardised assessment of paraffin-embedded tissues to record data using the Consortium to Establish a Registry of Alzheimer's Disease (CERAD) protocol [2]. CERAD data were augmented by a strategy for evaluating white matter lesions (WML) in the post mortem brain previously validated against histopathology [15]. Neuropathology was assessed without knowledge of clinical, interview, or RINI data. Acceptable inter-rater reliability (<5% with scores more than 1 grade difference) was achieved for cerebral cortical atrophy, NFT, amyloid plaques (diffuse and neuritic), Lewy bodies, and cerebral amyloid angiopathy by circulation of macroscopic brain photographs and microscopic slides.

### Statistical Methods

Sample characteristics were compared with the overall population using Chi-squared for association and Mann-Whitney for the median age of death. Unconditional logistic regression analysis examined the effect of neuropathological variables and age on dementia at death. Backwards stepwise logistic regression (*p = *0.1) assisted model selection. Interactions between pathologies were tested using likelihood ratio tests. The chosen final model best predicted dementia status at death for the least number of factors within the model. The method maximised the sensitivity and specificity using the predicted probabilities from the model, and minimised the number of individuals for whom prediction was not possible.

Using the model most likely to correctly predict dementia we estimated the partial “population attributable risk” (AR) at death, conditional on all other factors, from the adjusted logistic regression [16]. These AR estimates represent the amount of dementia at death in the sample determined by each factor in the model, relative to the reference category, using both the risk and the prevalence of that factor. The AR represents the proportion of dementia at death accounted for after elimination of that risk factor if the remaining distribution of the risk factors remains unchanged. Bootstrap confidence intervals were calculated [17]. The amount of missing data was small (<5%) except for brain weight (10%), macroscopic vascular disease (7%) and diffuse plaques in the entorhinal campus/hippocampus (6%). A sensitivity analysis of the model and PAR estimates was undertaken using multiple imputations using chain equations to impute the data for all individuals with unknown factors in the model process [18]. Ten imputation datasets were used. Analysis was undertaken using Stata software, version 9.2 (Stata Corp.) and the R software package (www.r-project.org).

## Results

### Representativeness of the Brain Donor Cohort

There were no significant differences between the donor cohort and all respondents who died with respect to sex and length in fulltime education (Table 1). Individuals who donated brain tissue were significantly older than all deaths in the population sample, and also than all individuals still alive on 1st August 2004. More donors were in the “manual” social class compared with all who died (*p = *0.03). There was no significant difference between the brain donor cohort and the CFAS baseline sample with respect to sex, social class, and length in fulltime of education.

**Table 1:** Demographic characteristics of baseline population, deaths, and donors (six centres).

Characteristics	Baseline *n = *18,248	Percent	IQR	Died but Not Donors *n = *11,465	Percent	IQR	Donors *n = *456	Percent	IQR	Donors Versus All Died but Not Donors
**Centre**										
Cambridge	2,601	14	—	1,465	13	—	96	21	—	—
Gwynedd	2,625	14	—	1,387	12	—	9	2	—	—
Liverpool	5,244	29	—	4,065	35	—	101	22	—	—
Newcastle	2,524	14	—	1,549	14	—	55	12	—	—
Nottingham	2,514	14	—	1,423	13	—	127	28	—	—
Oxford	2,740	15	—	1,526	13	—	68	15	—	—
**Sex**										
Men	7,625	42	—	5,164	45	—	188	41	—	—
Women	10,623	58	—	6,301	55	—	268	59	—	*p = *0.11
**Age group at baseline (y)**										
<74	8,231	45	—	3,557	31	—	100	22	—	—
75–84	6,982	38	—	5,159	45	—	215	47	—	—
85–94	2,849	16	—	2,573	22	—	135	29	—	—
>94	186	1	—	176	2	—	6	2	—	*P<*0.001
**Median age (y)**	70	—	67–74	79	—	73,84	81	—	76–86	—
**Education**										
Missing	566	3	—	483	4	—	32	6	—	—
0–9 y	11,985	66	—	7,812	68	—	302	67	—	—
>9 y	5,697	32	—	3,170	28	—	122	27	—	*p = *0.97
**Social class**										
Missing	1,371	8	—	1,035	9	—	57	13	—	—
Nonmanual	6,574	36	—	3,717	32	—	165	36	—	—
Manual	10,303	56	—	6,713	59	—	234	51	—	*p = *0.02
**Age group at death (y)**										
<74	—	—	—	1,187	10	—	43	27	—	—
75–84	—	—	—	4,601	40	—	125	50	—	—
85–94	—	—	—	4,782	42	—	229	13	—	—
>94	—	—	—	895	8	—	59	—	82–92	*P<*0.001
**Median age at death**	—	—	—	84	—	79,90	87	—	—	—

IQR, interquartile range.

### Prevalence of Neuropathologies in Old People

Neurodegenerative pathology in the cohort is shown in Tables 2 and 3. NFT were the most prevalent degenerative pathology in the hippocampus and entorhinal cortex (92%). In the neocortex NFT were less (52%), and neuritic plaques more (68%), prevalent. Neuronal loss in hippocampus and subcortical nuclei was reported in 40% and 60% of the sample, respectively. Lewy bodies were found in less than 10% of brains (mainly substantia nigra) reflecting the use of older techniques (hematoxylin–eosin or ubiquitin staining) rather than synuclein staining. These factors are shown in relation to dementia status in Tables 4 and 5. Neocortical, hippocampal, and entorhinal cortex pathology was more common in individuals with dementia. Vascular pathology was frequently observed (not demented 71%, demented 84%), most frequently as small vessel disease (SVD; not demented 60%, demented 77%; odds ratio [OR] for dementia 1.6, 95% confidence interval [CI] 0.9–2.8). This diagnosis of SVD is based exclusively on histological criteria. Periventricular white matter lesions (PVL) were common (not demented 87%, demented 95%, OR for dementia with severe PVL 4.3, 95% CI 1.9–9.8), though deep white matter lesions (DWMLs) were less common (not demented 60%, demented 73%, OR for dementia with severe DWML 3.3, 95% 1.6–6.8). A combined diagnosis including both WML and histological SVD was overrepresented among the demented group (32% versus 24%; OR for dementia 2.9, 95% CI 1.6–5.5). Cerebral amyloid angiopathy (CAA) was overrepresented in the demented group (34% versus 10%; OR for dementia 4.3, 95% CI 2.4–7.6). Neocortical and hippocampal atrophy were both common in individuals with dementia (75% and 67%), though less so in the nondemented (43% and 31%), and therefore have a very large association with dementia (severe atrophy OR>10).

**Table 2:** Number of individuals with neuropathology findings in medial temporal and neocortical regions.

Neuropathology	Severity	Hippocampus	Percent	Entorhinal	Percent	Frontal	Temporal	Parietal	Occipital	Overall	Percent
**Neuritic plaques**	**None**	191	42	178	39	185	171	194	200	143	31
	**Mild**	97	21	122	27	140	111	114	74	104	23
	**Moderate**	123	27	106	23	91	123	108	80	139	31
	**Severe**	39	9	41	9	37	50	38	23	70	15
	**Missing**	6	1	9	2	3	1	2	79	0	0
**Diffuse plaques**	**None**	203	45	142	31	134	130	151	143	115	25
	**Mild**	128	28	110	24	106	95	107	94	95	21
	**Moderate**	84	18	124	27	94	122	92	100	112	25
	**Severe**	16	4	51	11	103	92	88	37	120	26
	**Missing**	25	5	29	6	19	17	18	82	14	3
**Tangles**	**None**	51	11	39	9	298	230	295	299	216	47
	**Mild**	118	26	94	21	101	124	94	38	129	28
	**Moderate**	134	30	185	41	40	62	47	26	70	15
	**Severe**	147	32	128	28	11	37	11	9	39	9
	**Missing**	6	1	10	2	6	7	9	84	2	1
**Neuronal loss**	**None**	287	63	287	63	432	430	430	384	425	93
	**Mild**	67	15	53	12	10	12	7	3	19	4
	**Moderate**	39	9	46	10	0	0	0	0	0	0
	**Severe**	37	8	34	7	0	0	0	0	0	0
	**Missing**	26	6	36	8	14	14	19	69	12	3
**Lewy bodies**	**None**	432	95	409	90	433	424	428	380	425	93
	**Mild**	6	1	13	3	8	16	4	2	18	4
	**Moderate**	0	0	4	1	0	0	0	0	0	0
	**Severe**	0	0	1	0	0	0	0	0	0	0
	Missing	18	4	29	6	15	16	24	74	13	3

**Table 3:** Number of individuals with neuropathology findings in subcortical nuclei.

Neuropathology	Severity	Substantia Nigra	Nucleus Basalis	Raphé	Locus Ceruleus	Dorsal Vagus	Overall	Percent
**Plaques**	**None**	389	202	271	283	194	353	77
	**Mild**	2	30	8	1	0	36	8
	**Moderate**	0	13	0	0	0	13	3
	**Severe**	0	0	0	0	0	0	0
	**Missing/not measured**	65	211	177	172	262	54	12
**Tangles**	**None**	314	93	157	166	207	177	39
	**Mild**	89	104	78	105	11	122	26
	**Moderate**	13	49	52	42	0	77	17
	**Severe**	20	47	36	14	0	71	15
	**Missing**	20	163	133	129	238	9	2
**Neuronal loss**	**None**	217	205	298	216	230	183	40
	**Mild**	175	61	23	84	27	175	38
	**Moderate**	36	23	2	27	14	67	15
	**Severe**	16	6		11	3	25	6
	**Missing**	12	161	133	118	182	6	2
**Lewy bodies**	**None**	409	286	327	313	258	406	89
	**Mild**	19	10	2	15	7	20	4
	**Moderate**	11	1	0	9	10	18	4
	**Severe**	8	0	0	1	0	9	2
	**Missing**	9	159	127	118	181	3	1

**Table 4:** Number of individuals by neuropathology and dementia status at death.

Neuropathological Findings	Severity	No Dementia *n = *183	Percent	Dementia *n = *243	Percent	Uncertain *n = *30	Percent
**Neocortex: neuritic plaques**	**None**	83	45	47	19	13	43
	**Mild**	51	28	44	18	9	30
	**Moderate**	43	24	89	37	7	23
	**Severe**	6	3	63	26	1	3
**Neocortex: diffuse plaques**	**None**	54	30	38	16	9	30
	**Mild**	43	24	42	18	4	13
	**Moderate**	56	31	60	26	9	30
	**Severe**	28	15	91	39	8	27
	**Missing**	12	—	12	—	0	—
**Neocortex: NFT**	**None**	114	63	81	33	21	67
	**Mild**	59	33	63	26	7	22
	**Moderate**	8	4	60	25	2	11
	**Severe**	0	0	39	16	0	0
**Neocortex: atrophy**	**None**	101	57	57	25	15	50
	**Mild**	51	29	52	23	12	40
	**Moderate**	24	14	94	41	2	7
	**Severe**	1	1	28	12	0	0
	**Missing**	6	—	12	—	1	—
**Hippocampus:** **neuritic plaques**	**None**	106	58	67	28	18	60
	**Mild**	37	20	57	24	3	10
	**Moderate**	33	18	83	35	7	23
	**Severe**	6	3	31	13	2	7
	**Missing**	1	—	5	—	0	—
**Hippocampus:** **diffuse plaques**	**None**	101	56	89	40	13	43
	**Mild**	47	26	70	32	11	37
	**Moderate**	26	15	53	24	5	17
	**Severe**	5	3	10	5	1	3
	**Missing**	4	—	21	9	0	—
**Hippocampus:** **NFT**	**None**	34	19	12	5	5	17
	**Mild**	68	37	39	16	11	37
	**Moderate**	53	29	72	30	9	30
	**Severe**	27	15	115	48	5	3
	**Missing**	1	—	5	—	0	0
**Hippocampus: atrophy**	**None**	117	69	71	33	16	57
	**Mild**	33	19	47	22	9	32
	**Moderate**	17	11	78	36	2	7
	**Severe**	2	1	18	8	1	4
	**Missing**	14	—	29	—	2	2
Entorhinal cortex: neuritic plaques	**None**	100	55	61	26	17	57
	**Mild**	49	27	64	27	9	30
	**Moderate**	28	15	75	32	3	10
	**Severe**	5	3	35	15	1	3
	**Missing**	1	—	8	—	0	—
**Entorhinal cortex: diffuse plaques**	**None**	81	46	51	23	10	33
	**Mild**	42	24	58	26	10	33
	**Moderate**	44	25	73	33	7	23
	**Severe**	11	6	37	17	3	10
	**Missing**	5	—	24	—	0	—
**Entorhinal cortex: NFT**	**None**	27	15	7	3	5	17
	**Mild**	55	30	30	13	9	30
	**Moderate**	77	43	98	42	10	33
	**Severe**	22	12	100	43	6	20
	**Missing**	2	—	8	—	0	—
**Lewy bodies**	**—**	10	5	35	14	3	10
**Brain weight** kg – median	**—**	1.24	—	1.11	—	1.15	—
**Age at death (y)**	**<80**	57	32	25	10	7	23
	**80–89**	79	43	110	45	15	50
	**≥90**	47	26	108	44	8	27
**CAA**	**None**	131	73	102	43	23	77
	**Mild**	31	17	54	23	4	13
	**Moderate**	17	10	64	27	2	7
	**Severe**	0	0	17	7	1	3
	**Missing**	5	—	5	—	0	—
**Moderate/severe CAA**	**—**	17	10	81	34	3	13
Vascular disease							
**Any vascular disease**	**—**	122	71	196	84	24	83
**Haemorrhage**	**—**	9	5	9	4	1	4
**Infarct**	**—**	43	24	80	34	14	48
**Lacune**	**—**	30	17	62	26	7	24
**SVD**	**—**	104	60	178	77	18	64
Overall vascular pathology	**—**	18	—	—	—	—	—
**None**	**—**	50	29	38	17	5	18
**One of infarct/lacune/haemorrhage**	**—**	14	8	9	4	3	11
**SVD only**	**—**	56	33	76	34	8	29
**Multiple**	**—**	50	29	102	45	12	43
**Missing**	**—**	18	—	13	—	2	—
**Periventricular WML**	**None**	21	13	11	5	2	7
	**Mild**	95	58	100	44	19	66
	**Moderate/severe**	49	29	114	51	8	28
	**Missing**	19	—	17	—	1	—
**Deep subcortical WML**	**None**	71	40	62	27	12	41
	**Mild**	67	38	97	41	12	41
	**Moderate**	24	14	36	15	4	14
	**Severe**	14	8	39	17	1	3
	**Missing**	9	—	4	—	1	—
**Histological/imaging vascular disease**							
**None**	**—**	44	25	26	11	5	17
**Infarct or haemorrhage**	**—**	7	4	5	2	1	3
**Lacunes/SVD/DWML**	**—**	84	47	130	55	11	37
**Both**	**—**	43	24	77	32	13	43
**Missing**	**—**	5	—	5	—	0	—

**Table 5:** Unconditional logistic regression adjusted for age and multivariable analyses and estimated partial AR at death for dementia.

Neuropathological Findings	Age Adjusted Analysis			Multivariable Analysis		AR	
	OR	95% CI	*p*-Value	OR	95% CI	Percent	95% CI
**Age at death (y)**							
**<80**	1.0	**—**	—	1.0	—	—	**—**
**80–89**	3.2	1.8–5.5	—	2.5	1.1–5.8	8	0–16
**≥90**	5.2	2.9–9.4	<0.001	3.4	1.4–8.3	10	3–16
**Time since last interview (y)**	**—**	**—**	—	Not included	—	Not included	**—**
**<1**	1.0	**—**	—	—	—	—	**—**
**>1**	1.2	0.8–1.7	0.5	—	—	—	**—**
**Brain weight for sex (g)**							
**Low**	5.7	3.2–10	<0.001	4.1	1.9–9.2	12	5–19
**Average**	2.0	1.2–34	—	2.1	1.0–4.2	5	0–11
**High**	1.0	**—**	—	1.0	—	—	**—**
**Neuritic plaques in neocortex**							
**None**	1.0	**—**	—	1.0	—	—	**—**
**Mild**	1.2	0.7–2.2	—	1.0	—	—	**—**
**Moderate**	2.9	1.7–5.0	—	1.0	—	—	**—**
**Severe**	18.5	7.3–47	<0.001	9.7	2.1–43	8	3–14
**Diffuse plaques in neocortex**	**—**	**—**	—	Not included	—	Not included	**—**
**None**	1.0	**—**	—	—	—		**—**
**Mild**	1.4	0.7–2.5	—	—	—	—	**—**
**Moderate**	1.6	0.9–2.9	—	—	—	—	**—**
**Severe**	4.2	2.3–7.9	<0.001	—	—	—	**—**
**NFT in neocortex**							
**None**	1.0	**—**	—	1.0	—	—	**—**
**Mild**	1.3	0.8–2.1	—	1.0	0.5–1.8	—	**—**
**Moderate**	8.9	4.0–20	—	7.1	2.3–22	11	5–19
**Severe**	∞	**—**	<0.001	—	—	—	**—**
**Neuritic plaques in hippocampus**	**—**	**—**	—	Not included	—	Not included	**—**
**None**	1.0	**—**	—	—	—	—	**—**
**Mild**	1.9	1.1–3.3	—	—	—	—	**—**
**Moderate**	3.5	2.1–5.9	—	—	—	—	**—**
**Severe**	8.5	3.2–22	<0.001	—	—	—	**—**
**Diffuse plaques in hippocampus**	**—**	**—**	—	Not included	—	Not included	**—**
**None**	1.0	**—**	—	—	—	—	**—**
**Mild**	1.5	0.9–2.5	—	—	—	—	**—**
**Moderate**	2.0	1.1–3.5	—	—	—	—	**—**
**Severe**	2.2	0.7–7.2	0.06	—	—	—	**—**
**Tangles in hippocampus**	**—**	**—**	—	Not included	—	Not included	**—**
**None**	1.0	**—**	—	—	—	—	**—**
**Mild**	1.3	0.6–2.8	—	—	—	—	**—**
**Moderate**	2.7	1.2–5.9	—	—	—	—	**—**
**Severe**	8.4	3.7–19	<0.001	—	—	—	**—**
**Neuritic plaques in the entorhinal cortex**	**—**	**—**	—	Not included	—	Not included	**—**
**None**	1.0	**—**	—	—	—	—	**—**
**Mild**	1.9	1.1–3.1	—	—	—	—	**—**
**Moderate**	3.7	2.1–6.5	—	—	—	—	**—**
**Severe**	11.1	4.0–30	<0.001	—	—	—	**—**
**Diffuse plaques in the entorhinal cortex**	**—**	**—**	—	Not included	—	Not included	**—**
**None**	1.0	**—**	—	—	—	—	**—**
**Mild**	2.2	1.3–3.8	—	—	—	—	**—**
**Moderate**	2.6	1.5–4.4	—	—	—	—	**—**
**Severe**	5.5	2.5–12	<0.001	—	—	—	**—**
**Tangles in entorhinal cortex**	**—**	**—**	—	Not included	—	Not included	**—**
**None**	1.0	**—**	—	—	—	—	**—**
**Mild**	1.7	0.6–4.5	—	—	—	—	**—**
**Moderate**	3.4	1.4–8.6	—	—	—	—	**—**
**Severe**	12.7	4.8–34	<0.001	—	—	—	**—**
**CAA**							
**None**	1.0	**—**	—	1.0	1.0	—	**—**
**Mild**	1.9	1.1–3.3	—	1.8	0.8–3.8	2	0–6
**Moderate**	4.0	2.2–7.4	—	2.9	1.2–6.8	5	1–10
**Severe**	∞	**—**	<0.001	2.9	1.2–6.8	5	1–10
**Moderate/severe CAA**	**—**	**—**	—	Not included	—	Not included	**—**
**No**	1.0	**—**	—	—	—	—	**—**
**Yes**	4.3	2.4–7.6	<0.001	—	—	—	**—**
**Lewy bodies**							
**No**	1.0	1.0	—	1.0	—	—	**—**
**Yes**	3.2	1.5–6.9	<0.003	3.5	1.3–9.3	3	1–7
**Vascular disease (VD)**	**—**	**—**	—	Not included	—	Not included	**—**
**None**	1.0	**—**	—	—	—	—	**—**
**One of infarct/lacune/haemorrhage**	0.7	0.3–1.9	—	—	—	—	**—**
**SVD only**	1.6	0.9–2.8	—	—	—	—	**—**
**Multiple**	2.5	1.4–4.3	<0.004	—	—	—	**—**
**Overall vascular pathology**							
**None**	1.0	**—**	—	1.0	—	—	**—**
**Infarcts/haemorrhage**	1.0	0.3–3.8	—	2.4	0.4–12	—	**—**
**SVD/WML/lacunes**	2.5	1.4–4.4	—	3.7	1.5–9.6	12	3–19
**Both**	2.9	1.6–5.5	<0.003	4.8	1.9–12	9	3–15
**Periventricular WML**	**—**	**—**	—	Not included	—	Not included	**—**
**None**	1.0	**—**	—	—	—	—	**—**
**Mild**	2.0	0.9–4.5	—	—	—	—	**—**
**Moderate/severe**	4.3	1.9–9.8	<0.001	—	—	—	**—**
**Deep subcortical WML**	**—**	**—**	—	Not included	—	Not included	**—**
**None**	1.0	**—**	—	—	—	—	**—**
**Mild**	1.6	1.0–2.6	—	—	—	—	**—**
**Moderate**	1.6	0.9–3.1	—	—	—	—	**—**
**Severe**	3.3	1.6–6.8	<0.01	—	—	—	**—**
**Hippocampal atrophy**							
**None**	1.0	1.0	—	1.0	1.0	—	**—**
**Mild**	2.2	1.3–3.9	—	1.8	0.9–3.7	2	0–6
**Moderate**	6.9	3.7–13	—	3.4	1.5–7.5	8	2–15
**Severe**	11.1	2.5–50	<0.001	3.4	1.5–7.5	8	2–15
**Neocortical atrophy**	**—**	**—**	—	Not included	—	Not included	**—**
**None**	1.0	1.0	—	—	—	—	**—**
**Mild**	1.6	1.0–2.8	—	—	—	—	**—**
**Moderate**	5.9	3.3–10	—	—	—	—	**—**
**Severe**	37.8	5.0–29	<0.01	—	—	—	**—**

The multivariable logistic regression (Table 5) showed independent association with dementia status for: moderate and severe NFT in the neocortex; severe neuritic plaques in the neocortex; cerebral angiopathy; combined vascular disease; SVD; PVL; Lewy bodies; hippocampal atrophy; brain weight and age. All respondents with severe NFT in the neocortex (39 individuals, 16% of the demented group) were demented giving an infinite odds ratio for dementia risk and representing a perfect predictor (sufficient but not necessary). Seventy individuals had some missing neuropathology measures and were initially excluded from the modelling. Individuals whose missing data did not affect their predicted dementia status were included in the summary (48 of 70). The final prediction was therefore from a model using 404 brains (89%), which represent 4% of all the deceased respondents in CFAS.

The multivariable model (Table 5) correctly predicted dementia status in 80% (404 individuals) of the 426 with known dementia status (sensitivity, 71%, 95% CI 64–76; specificity, 92%, 95% CI 87–96; receiver operating characteristic [ROC] 0.86). Substantial neuropathology without a dementia diagnosis was found in 13 individuals (M∶F 6∶7; age at death, 81–102 y). This group includes two individuals with life-long low cognition confirmed by RINI interview. The remaining 11 had an Mini-Mental Status Examination (MMSE) >18 (three with MMSE >26) when they were last measured, including five (45%) who had a RINI. Median time from interview to death in those without a RINI interview was 12 mo. Only two had symptomatic cognitive impairment, but not consistently, and one had depression. The pathologies exhibited by these individuals were SVD (*n = *11), low brain weight (*n = *8), atrophy (*n = *7), severe plaques (*n = *5), and moderate NFT (*n = *4).

Conversely 68 individuals (M∶F 24∶44; age at death, 71–103 y) had a dementia diagnosis before death but showed only modest neuropathology. Sixty (88%) showed moderate or severe cognitive impairment before death. Of the other eight all had died at least 15 mo after the last interview and dementia was confirmed by RINI (*n = *6) or death certificate (*n = *3). The neuropathology was generally mild and included Lewy bodies (*n = *4). Two individuals had severe atrophy of the hippocampus. Neuropathology in the brainstem, not included in the model, was present in 36 individuals (NFT, *n = *24; plaques, *n = *4; neuronal loss, *n = *15). These factors did not improve the overall model when tested across all individuals. Other neuropathological findings in these individuals include Progressive Supranuclear Palsy, hippocampal hypoxic injury, head injury, and mesial temporal sclerosis. The outcome of interest in this analysis was dementia and it therefore does not address cognitive impairment short of dementia in which the factors reported here would also be expected to play a role.

### AR of Dementia for Pathological Features

The risk of dementia associated with specific thresholds of pathology is shown in Table 5. Each estimated AR at death adjusts for all others such that 96% of the overall risk is explained. Nearly 20% of this risk is due to the effect of age. Factors conveying more than 8% each of the dementia risk were: NFT in the neocortex; age; neuritic plaques; SVD; moderate/severe atrophy; low brain weight. Alzheimer pathologies together (plaques, tangles, and CAA) account for ∼25% of dementia risk, and vascular pathologies ∼21%. Other neuropathological factors each convey between 2%–5% of the risk.

### Neuropathology in the Nondemented

Many nondemented individuals manifest “high risk” pathologies. A moderate NFT score in the neocortex is rare (4%), and a severe NFT score absent, but multiple vascular disease (24%) and SVD (47%) are common. Neuropathological factors, age, and brain weight only account for 34% of the variability within the model, despite high estimates of AR. This apparent anomaly underscores their relatively poor predictive value in making a diagnosis of dementia.

### Prediction from Neuropathology Alone

Univariate modelling of the relationship between dementia and neuropathological findings, excluding age and brain weight, showed a large additional risk associated with having NFT in the hippocampus. However this adjusted out in multivariable analysis. The model based on neuropathology data alone has higher sensitivity (83%), but lower specificity (76%). From the 399 in this model, 80% were correctly classified as either demented or not. The AR at death for neuropathological features was modified only slightly by excluding age and brain weight. The major contributor to dementia risk remained NFT (28%; neocortical, 14%; hippocampal, 14%). Atrophy (20%) and CAA (11%) were more important. Vascular factors (17%), neocortical plaques (7%), and Lewy bodies (4%) remained the same. No interactions were detected.

### Sensitivity Analysis

Imputation of variables with missing data was used to test the robustness of the model against both missing outcome variables (30 individuals whose dementia status was not coded) and pathology variables. The multivariable modelling after imputation showed few differences from the original model in Table 5. The neuropathology factors chosen to be represented in the model were checked using ten imputation datasets. Factors associated with dementia were remarkably stable within each imputation dataset. The only factors that appeared to differ were whether Lewy bodies (excluded from five datasets), severe plaques (excluded from four datasets), age (excluded from two datasets), and hippocampal atrophy (excluded from one dataset) should be included in the model. Two factors that were not previously in the models became important: NFT in the hippocampus and neuronal loss in the brainstem. Neuronal loss in the brainstem appeared important in individuals previously misclassified, but did not improve the model using the original data where there was missing data in the covariates and outcome variable. The full model with all these factors is shown in Table 6. The estimations of AR at death were very similar for the imputation datasets. The inclusion of brain stem neuronal loss (AR 13%) and NFT in the hippocampus (AR 5%) emerged from small reductions (1%) in the majority of factors though hippocampal atrophy (8% to 4%) and old age (11% to 8%) were more affected. Analysis adjusting for demographic differences between the brain donor cohort and the rest of the population that died showed only slight change in AR at death for factors most associated with older age (old age, atrophy, and neocortical NFT), whilst vascular disease, low brain weight, plaques, and CAA all showed small increases (1%–2%). Only low brain weight (from 11% to 18%) and atrophy (from 8% to 2%) were affected by the age difference between the donor cohort and all those who died in the population. A further sensitivity analysis only in those assessed less than 1 y prior to death was undertaken and all associations increased in strength, suggesting any bias is conservative, and all AR estimates were consistent with the confidence intervals presented.

**Table 6:** Sensitivity analysis: Imputation models.

Neuropathological Findings	Multivariable Model Original		Imputed		Imputation Model			
	OR	95%CI	OR	95% CI	OR	95%CI	AR	95% CI
**Age at death**								
**<80 y**	1.0	**—**	1.0	**—**	1.0	**—**	**—**	**—**
**80–89 y**	2.5	1.1–5.8	2.3	1.1–4.8	2.1	1.0–4.5	7	1–14
**≥90 y**	3.4	1.4–8.3	4.3	2.0–9.5	4.2	1.8–9.6	8	2–16
**Brain weight for sex**								
**Low**	4.1	1.9–9.2	4.3	2.0–9.2	4.3	1.9–9.6	12	5–19
**Average**	2.1	1.0–4.2	1.8	0.9–3.4	1.7	0.9–3.4	4	0–9
**High**	1.0	**—**	1.0	**—**	1.0	**—**	**—**	**—**
**Neuritic plaques in neocortex**								
**None or mild**	1.0	**—**	1.0	**—**	1.0	**—**	**—**	**—**
**Moderate or severe**	9.7	2.1–43	4.4	1.6–12	3.9	1.3–11	7	3–17
Tangles in neocortex								
**None**	1.0	**—**	1.0	**—**	1.0	**—**	**—**	**—**
**Mild**	1.0	0.5–1.8	1.0	0.6–1.8	0.7	0.4–1.4	**—**	**—**
**Moderate or severe**	7.1	2.3–22	6.3	2.6–15	4.6	1.8–12	10	5–17
**Tangles in hippocampus**								
**None or mild**	**—**	**—**	**—**	**—**	1.0	**—**	**—**	**—**
**Moderate or severe**	Not included	**—**	Not included	**—**	1.8	1.0–3.3	5	0–12
**CAA**								
**None**	1.0	**—**	1.0	**—**	1.0	**—**	**—**	**—**
**Mild**	1.8	0.8–3.8	1.7	0.9–3.3	1.5	0.7–3.0	**—**	**—**
**Moderate or severe**	2.9	1.2–6.8	3.5	1.7–7.4	3.8	1.7–8.2	4	0–9
**Lewy bodies**								
**No**	1.0	**—**	1.0	**—**	1.0	**—**	**—**	**—**
**Yes**	3.5	1.3–9.3	3.7	1.6–8.9	2.2	0.9–5.7	2	0–5
**Overall vascular pathology**								
**None**	1.0	**—**	1.0	**—**	1.0	**—**	**—**	**—**
**Infarcts/haemorrhage**	2.4	0.4–12	1.9	0.3–11	1.4	0.2–9.4	**—**	**—**
**SVD/DWML/lacunes**	3.7	1.5,9.6	3.1	1.4–6.9	3.3	1.4–7.5	**—**	**—**
**Both**	4.8	1.9–12	4.0	1.8–9.2	4.2	1.8–9.9	20	8–33
**Hippocampal atrophy**								
**None**	1.0	**—**	1.0	1.0	1.0	**—**	**—**	**—**
**Mild**	1.8	0.9,3.7	1.5	0.8–2.9	1.3	0.6–2.6	**—**	**—**
**Moderate**	3.4	1.5–7.5	2.8	1.4–5.6	1.9	0.9–4.2	4	0–10
**Severe**	**—**	**—**	**—**	**—**	**—**	**—**	**—**	**—**
**Brainstem neuronal loss**								
**None**	**—**	**—**	**—**	**—**	1.0	1.0	**—**	**—**
**Mild**	**—**	**—**	**—**	**—**	2.7	1.5–4.9	6	1–12
**Moderate**	**—**	**—**	**—**	**—**	3.3	1.4–8.0	7	0–13
**Severe**	Not included	**—**	Not included	**—**	9.9	1.8–54	7	0–13

## Discussion

MRC CFAS shows that it is possible to set up and sustain a brain donation programme from a geographically dispersed, population-based study, which is not biased in terms of gender, social class, education, institutionalisation, or access to health care. The resulting brain donor sample is of sufficient size to generate meaningful estimates of AR at death associated with specific pathologies and contributes significantly to understanding the pathobiology of dementia on the basis of “epidemiological neuropathology.” It also allows the separation of factors that might be amenable to modification from others that may not. The main contributors to AR at death for dementia in MRC CFAS were age (18%), small brain (12%), neocortical neuritic plaques (8%) and neurofibrillary tangles (11%), small vessel disease (12%), multiple vascular pathologies (9%), and hippocampal atrophy (10%). Other significant factors include cerebral amyloid angiopathy (7%) and Lewy bodies (3%).

Earlier CFAS analysis showed that Alzheimer pathology and vascular disease are frequently found in both demented and nondemented people [7]. In the present more detailed analysis, with larger numbers, a moderate or severe neocortical NFT score emerged as the best pathological discriminator between the demented and nondemented groups. SVD emerged as an independent contributor to dementia risk in keeping with the evidence that SVD is the substrate for “subcortical vascular dementia” and contributes a major part of the burden of vascular cognitive impairment in the population [19]. We included deep subcortical WMLs within the vascular disease variable on the basis of evidence that they arise through vascular mechanisms [20]. The estimates of AR at death reveal the relative importance of conventional pathological measures at the population level and show a range of pathological features contributing independently to dementia.

The major independent effects of age and relative low brain weight are interesting. The findings imply that other factors, not captured in this standardised approach to pathological analysis, are determinants of cognitive trajectory in older people. These may include synaptic integrity and the concentrations of peptide oligomers [21],[22] but also interindividual variation in diverse factors that determine the neurobiological basis of “brain reserve,” both innate (synaptic and neuronal density achieved into adult life, potential for neurogenesis, synaptic plasticity) and acquired (educational attainment, sustained intellectual, social, or physical activity in mid-life and old age) [23].

### Limitations of the Study

In these six population samples from England and Wales the baseline response rate was good and unlikely to have been severely biased. Considerable attrition over time determines that those who remain in the study tend to have been younger and fitter at enrolment [24]. The cohort reported here is based on individuals who were selected for more detailed assessment at the baseline and year 2 waves. Selection to this group is weighted towards the cognitively impaired, but with random selection from the full population, and created an older sample than the remainder of the baseline sample who died within the study period. Causes of death were similar in the two groups. Because the characteristics of both samples are known, a sensitivity analysis backweighting for this process (and for biases arising from selection into the neuropathology cohort) adequately adjust for these sampling effects, though not for unknown biases.

The number of interviews achieved for each individual during the study varied (96% had at least two and 30% had five or more). The AGECAT algorithm, applied to the data at last interview, has been validated against clinical diagnoses and shown to be comparable to Diagnostic and Statistical Manual of Mental Disorders, Third Edition, Revised (DSM-IIIR) [25]. Fieldwork interviews were rarely started in acutely ill individuals so that diagnoses are unlikely to be influenced by confusional states. Although dementia on death certificates, insensitive but highly specific, was used to find incident dementia between last interview and death it was not used to indicate that an individual was not demented [26],[27]. Clinical judgements from informant reports were based on DSM-III-R, consistent with the previous validation studies using the GMS instrument. The extent of misclassification that would be necessary to create the findings observed here would need to be extreme and, when including only those with most recent interview data, the results are not affected.

Our previous report, clinically allocating differential diagnoses with a predominance of mixed pathology, remains robust [7]. The factors identified in this analysis coexist and may interact mechanistically. Our analysis does not allow us to elucidate causal directions, which are better investigated using longitudinal analysis and in experimental work. A formal analysis of interactions between vascular and degenerative pathologies will be reported separately but the models here did not reveal interactions. The low prevalence of Lewy bodies in this sample (∼10%), reflects methods that were not optimised for the detection and screening of α-synucleinopathy because the neuropathology protocol predates the recognition of dementia with Lewy bodies, and the discovery of α-synuclein in Lewy bodies. Recent data on a subset of this cohort show synucleinopathy in 37% but no strong association with dementia [28]. Our data on brain weight are based on comparisons within sex but this measure does not distinguish atrophy from innate smallness, an issue that can only be addressed by systematic measurement of total cranial volume. Nor does it distinguish the contribution of vascular and neurodegenerative processes, or other correlates such as synaptic and dendritic loss that were not routinely measured in the pathology protocol. Standardised and validated assessment of vascular pathology is also needed in studies of the pathological correlates of dementia [29]. Perhaps the greatest difficulty in interpreting these data is that they derive from individuals who have died. People with dementia live for a variable length of time during which burdens of neuropathology are assumed to change. To extrapolate from this sample to an equivalent cross section of the living older population is problematic but, in the absence of methods to achieve in vivo measurement of all pathologies, this is the closest estimate it is currently possible to produce. In due course these data can be combined with modelling of in vivo population pathology derived from techniques to assess vascular and neuropathological changes (e.g., amyloid positron emission tomography [PET] scans).

The pathological features that are associated with dementia in this analysis are well supported by data from other large community-based and population-based studies. There is general agreement from studies of older people in the UK and the US that dementia is predominantly associated with mixed vascular and Alzheimer lesions together with other contributions of lesser degree (e.g., synucleinopathy) [30]–[32]. Those studies also contribute important insights into the potential interactions of vascular and degenerative pathologies that are not dealt with in the present analysis [30]–[35]. Some studies have emphasised the significance of microscopic infarcts compared to macroscopic infarcts in explaining the relationship between pathology and dementia [30],[32],[34], whereas others have not demonstrated an independent association of dementia risk with microscopic infarction [31],[35]. In the present analysis we did not treat microinfarcts as a single pathological variable. Rather we chose to incorporate them into a global assessment of significant intrinsic SVD that also included microscopic evidence of severe arteriolar sclerosis and the presence of severe white matter attenuation. The Adult Changes in Thought study (ACT) has estimated the OR for dementia associated with Lewy bodies to be 5.1 (95% CI 1.37–18.96) on the basis of α-synuclein immunocytochemistry compared to 3.5 (95% CI 1.3–9.3) in this study using a less reliable method of detection. The estimates of AR at death for Lewy body pathology are 10% in ACT and 3% in MRC CFAS as reported here. In a subgroup of this CFAS cohort we demonstrated synucleinopathy in 37% of donated brains [28]. Other large cohorts have reported no clear predictive relationship between Lewy body pathology and dementia [36]. Interpretation of data on Lewy body pathology in published multivariable analyses is further complicated by the recent recognition of “amygdala predominant disease” that may not be reliably detected using some screening protocols. Another pathology recently emphasised in older people is hippocampal sclerosis (HS), which has been shown to contribute a relative risk for dementia of 2.43 (95% CI 1.01–5.85). This is a microscopic diagnosis that was not included as a separate variable in our data. While we did include macroscopic hippocampal atrophy, and found that it contributes 10% of the AR at death for dementia, it is important for future studies to determine the correlation between macroscopic changes and the microscopic features of HS, which are also not yet the subject of diagnostic consensus or interlaboratory validation.

The present study supports the view that interventions that modify neuropathology related to dysmetabolism of specific proteins (βA4, tau) have the potential to impact on the population burden of dementia. In the context of presymptomatic treatment many individuals without risk of developing dementia would also be treated unless the predictive ability of clinical tests improves dramatically. However the estimates in this analysis indicate that individual pathologies contribute only modestly to the overall risk of dementia and emphasise the need to develop a range of protective strategies. Other factors, potentially less amenable to intervention play a role including age, and underlying innate or acquired factors relating to brain reserve, which, along with the effects of multiple pathological comorbidities, all play a part in the manifestation of dementia at the level of the population as a whole.

## Abbreviations

AR: attributable risk
CAA: cerebral amyloid angiopathy
CI: confidence interval
DWML: deep white matter lesion
GMS: geriatric mental state
NFT: neurofibrillary tangles
OR: odds ratio
RINI: retrospective informant interview
SVD: small vessel disease
WML: white matter lesion
